# People who seem disgusting seem more immoral

**DOI:** 10.3389/fpsyg.2024.1395439

**Published:** 2024-05-23

**Authors:** Sean M. Laurent, Jieming Li

**Affiliations:** ^1^Department of Psychology, University of Illinois at Urbana-Champaign, Champaign, IL, United States; ^2^Department of Psychology, The Pennsylvania State University, University Park, PA, United States

**Keywords:** disgust, emotion, moral judgment, moral character, punishment, person perception

## Abstract

Despite unresolved questions about replicability, a substantial number of studies find that disgust influences and arises from evaluations of immoral behavior and people. Departing from prior emphases, the current research examines a novel, related question: Are people who are viewed as disgusting (i.e., people whose habits seem disgusting) perceived as more immoral than typical or unusual people? Four experiments examined this, also exploring the downstream impacts of moral character judgments. Adults who seemed disgusting were regarded as more immoral for purity and non-purity violations (Experiment 1) and less praiseworthy for prosocial acts (Experiment 2). In Experiment 3, an 8-year-old with typical (but seemingly disgusting) habits was rated as “naughtier” and likelier to misbehave than an atypical child who loved vegetables and disliked sweets. Experiment 4 revealed how, when no behavioral information is available, beliefs about target disgust influence beliefs about future behavior, helping explain why seemingly disgusting targets are viewed as more immoral, but not always more punishable for their bad behavior.

## 1 Introduction

From an evolutionary perspective, disgust is a useful emotion that helps people avoid disease-causing pathogens (Curtis et al., 2011). Disgust also serves important psychological functions, helping people navigate behaviors related to sexuality, mate choice, and morality (Tybur et al., 2013, see also Watanabe and Laurent, 2020), suggesting that disgust and moral judgment have reasons to be linked (Chapman et al., 2009). Starting decades ago, a substantial amount of research effort has gone into uncovering whether this relationship exists. Resultant work has found that moral violations not only induce disgust (e.g., Rozin et al., 1999) but that disgust can itself amplify judgments of moral violations (Schnall et al., 2008; Horberg et al., 2009; Eskine et al., 2011; Russell and Giner-Sorolla, 2013; Olatunji et al., 2016), or even lead non-moral actions to be moralized (e.g., Prinz, 2006).

Although research has supported the idea that incidental disgust influences moral judgments, it is unclear whether these effects are replicable, unique to disgust (vs. other emotions like anger), or able to be found at all (e.g., Ghelfi et al., 2011; Cheng et al., 2013; Royzman et al., 2014; Johnson et al., 2016; Jylkkä et al., 2020; Białek et al., 2021, for a review, see Piazza et al., 2018). For example, a meta-analysis examining the effects of experimental manipulations of incidental disgust on moral judgments revealed that a small overall effect size was not significantly different from zero after controlling for publication bias (Landy and Goodwin, 2015, *cf.* Schnall et al., 2015).

Despite open questions regarding the relationship of incidental disgust to moral judgment, other possibilities remain for how morality and disgust might be associated (e.g., Pizarro et al., 2011). For example, work has shown that *feeling* disgusted or having higher trait-level disgust sensitivity can influence moral judgments (e.g., Jones and Fitness, 2008; Horberg et al., 2009; Chapman and Anderson, 2014; Białek et al., 2021). Similarly, although the extent of harmfulness vs. disgust might better explain the moralization of some behaviors (Royzman et al., 2014), and anger rather than disgust might be the predominant response when disgusting aspects of certain behaviors are excluded from stimuli (Kayyal et al., 2015), certain classes of immoral and norm-violating acts have been explicitly tied to disgust responses (e.g., Rozin et al., 1999; Horberg et al., 2009; Giner-Sorolla and Chapman, 2017; Andersson et al., 2024). These findings suggest again that disgust has a role to play in moral judgments in shaping responses to particular types of moral violations.

A further possibility for a role of disgust involves perceptions of moral character. Indeed, perceiving that a social target has an immoral character can induce feelings of disgust (Giner-Sorolla and Chapman, 2017; Giner-Sorolla et al., 2018). Moreover, purity-related (vs. non-purity-related) moral violations, theoretically linked to disgust responses (Rozin et al., 1999), are more likely to lead to inferences about moral character (Chakroff and Young, 2015; Sabo and Giner-Sorolla, 2017, but see Gray et al., 2014; Royzman et al., 2014), perhaps because disgust is less influenced than anger by external justifications (Russell and Giner-Sorolla, 2013). Consistent with this, although moral judgments like blame are highly sensitive to intent (e.g., Malle et al., 2014), purity violations appear to rely less on this mental state (Young and Saxe, 2011).

These findings suggest that the morality-disgust link may reside less in particular behaviors or in incidental experiences of disgust and more in how people think about and essentialize others. As recent work has shown, alongside other dimensions such as warmth and competence (Fiske, 2018), judgments regarding moral character are central to person perception (e.g., Pizarro and Tannenbaum, 2011; Goodwin et al., 2014; Goodwin, 2015). That is, people not only evaluate others’ behaviors or the consequences of their behaviors but also the *character* of people who behave in morally relevant ways, and these evaluations can inform future moral judgments (Pizarro and Tannenbaum, 2011). Thus, if some actions that are viewed as inherently disgusting by most people tend to promote inferences about poor moral character, it is also plausible that certain types of non-moral but seemingly *disgusting* (at least to some) behavioral habits will also promote inferences about moral character. Consistent with this, recent work has shown that dirty people are targets of discrimination (e.g., reduced trust relative to clean people) in both adults and children, even when a target’s state of dirtiness is not their fault (Helzer and Pizarro, 2011; Rottman et al., 2020). If true, this suggests potential real-world consequences. In everyday situations, being viewed as disgusting in some way (e.g., being caught nose-picking) could influence the perception of trustworthiness or mate fitness. As another more high-stakes example, if criminal prosecutors or plaintiffs can successfully paint a criminal or civil defendant as disgusting in some way, this might negatively influence the perception of guilt or result in higher punitive damages awarded.

This central question led us to examine a novel hypothesis in four experiments: That people with seemingly disgusting (vs. neutral or unusual, but not perceived as disgusting) habits would be perceived as more immoral. Experiment 1 examined this hypothesis for targets who committed purity and non-purity violations. Experiment 2 explored judgments following both moral violations and prosocial behaviors. Experiment 3 tested whether effects would emerge for an 8-year-old target whose potentially disgusting habits are relatively normative. Finally, Experiment 4 explored how expectations for behavior are swayed by the perceived disgustingness of a target and how this can lead to counterintuitive evaluations of behavior and punishment.

These experiments, for which data collection began on November 1, 2019, and ended on July 2, 2020, were not preregistered. Completely deidentified data are available at the Open Science Framework,^1^ along with a Supplemental Online Materials (SOM) document that describes all exclusions and criteria, verbatim instructions and methods for all experiments, supplemental analyses, and analysis syntax. All manipulations and measures are disclosed. Assignment to experimental conditions was always random. Sample sizes (Studies 1, 3, and 4) and stopping rules (Study 2) were determined *a priori* with the goal of being able to find medium-sized or smaller effects (*d* = 0.50), and no data were analyzed until all data were collected. Sensitivity analyses showed that sample sizes were sufficient to find effect sizes with 80% power (*α* = 0.05, two-tailed) for a two-group comparison of *d* = 0.36 (Experiment 2) or smaller.

## 2 Experiment 1

### 2.1 Methods

#### 2.1.1 Participants

Participants (*n* = 275; *M*_age_ = 36.59, *SD* = 11.25; 113 females, 160 males, two non-binary or preferred to not report) in Experiment 1 (and Experiments 3 and 4) were a convenience sample recruited from Amazon Mechanical Turk (AMT) and paid a small fee for participating. See Supplementary Table S1 in the SOM for information about exclusions. Retained participants self-identified as White/European American (*n* = 205), Black/African American (*n* = 21), Asian/Asian American (*n* = 22), Hispanic/Latino(a) (*n* = 20), and mixed/other (*n* = 7). On a scale measuring ideology that ranged from 1 = “extremely liberal” to 7 = “extremely conservative,” average ratings were near the midpoint, *M* = 3.41, *SD* = 1.69.

#### 2.1.2 Procedure and measures

The consent process was the same for all studies reported here. Participants completed all studies on their own devices (e.g., a computer) and were presented with an informed consent form. After reading the form, participants were instructed to click “continue” if they affirmatively consented to complete the study, and they were informed that they could remove their consent at any time by simply closing their browser window. After consenting to participate and being instructed as to the nature of the task they would complete, participants read one of four short descriptions of “John.” Participants were randomly assigned to read that John’s habits were either those we expected people to view as disgusting (e.g., snacking on dead skin from his feet while watching TV) or mundane (e.g., snacking on chips while watching TV). Participants were also randomly assigned to read that John committed one of two violations [i.e., “public” masturbation (the target masturbated in the restroom stall of a convenience store; thus, they were in public but were in a relatively private space) or shoplifting in a convenience store; see SOM] and responded (1 = not at all, 7 = extremely) to measures asking about the target’s disgustingness (disgusting, sick; *r* = 0.76), immoral character (immoral, evil; *r* = 0.68), the immorality of his behavior (wrong, immoral, bad, forbidden; *α* = 0.94), and his deservingness of punishment, if caught (1 *=* no punishment at all, 7 = a lot of punishment). We note that behavior disgustingness was measured but excluded to reduce common method variance. Finally, participants provided demographic information.

### 2.2 Results

**Figure 1:**
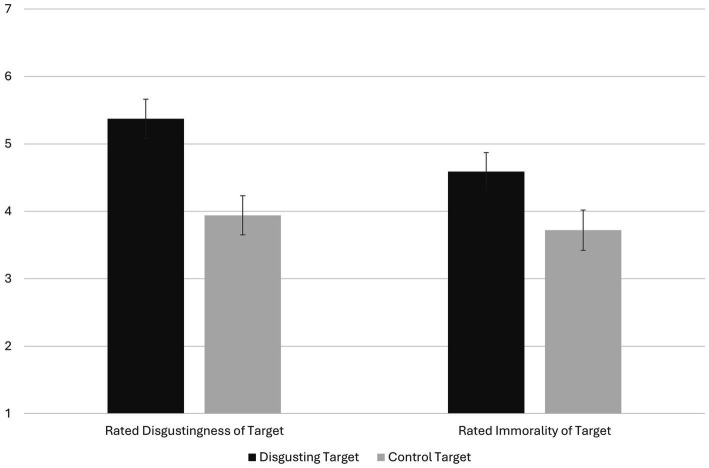
The effect of target condition on targets’ rated disgustingness and immorality in Experiment 1. Mean differences for both depicted variables were significant, *p*s < 0.001. Error bars are 95% confidence intervals. Variables are on seven-point scales.

The degrees of freedom for all ANOVA tests was 1, 271. See the SOM for non-essential information from all experiments (e.g., descriptive statistics involving method factors results from tests of method factors that did not change the substantive interpretation of primary effects). Note that in the results/discussion sections of all experiments, we refer to “disgusting” targets to be concise. More precisely, they are targets we expected people to rate as more disgusting than control targets. Checking the manipulation, the disgusting target (*M* = 5.37, 95% CI = 5.08, 5.66) was rated as more disgusting than the control target (*M* = 3.94; 95% CI = 3.65, 4.24), *F* = 45.81, *p* < 0.001, *d* = 0.81 (see Figure 1). Violation type (*p* = 0.616) and interaction with target type (*p* = 0.463) were not significant predictors of target disgust ratings. As hypothesized, the disgusting target (*M* = 4.59; 95% CI = 4.31, 4.88) was rated as more immoral than the control target (*M* = 3.72; 95% CI = 3.42, 4.01), *F* = 19.37, *p* < 0.001, *d* = 0.51 (Figure 1). Targets were also rated as more immoral following the non-purity (vs. purity) transgression (*p* < 0.001), but the interaction with target habits was not significant, *p* = 0.436. Although shoplifting was rated as more immoral and punishable than public masturbation (*p*s < 0.001), the total effects of the target condition on behavior immorality and punishment were not significant (*p*s = 0.199 and 0.628), nor were the interactions, *p*s = 0.583 and 0.248.

These latter findings were perplexing because of the strong positive correlations between immoral character judgments and behavior immorality and punishment (*r*s > 0.62) suggested that these latter variables would be similarly impacted by condition. Thus, in addition to the planned analysis below, several additional exploratory analyses were performed.

We first examined whether the effects of condition (analyses controlled for violation type) on character judgments were mediated by perceived target disgustingness (condition → target disgustingness → target immorality). The indirect effect was significant (*b* = 1.20, 95% = 0.84, 1.56), suggesting that because of perceived disgustingness, the disgusting (vs. average) target was viewed as having a more immoral character. However, the direct effect of condition on character was also significant, but it was reversed in sign, suggesting the disgusting (vs. average) target was perceived as *less* immoral (see the SOM for details of this analysis and others noted below) after controlling the effects of perceived disgustingness. This prompted several additional exploratory models to assess disgust and immoral character as mediators between condition and behavior immorality and punishment. In each model, significant indirect effects in line with hypotheses were countered by significant direct effects opposite in sign. We speculate about the reason for this (and similar findings here and across studies) in the Discussion section of Experiment 4 and in the General Discussion section.

**Figure 2:**
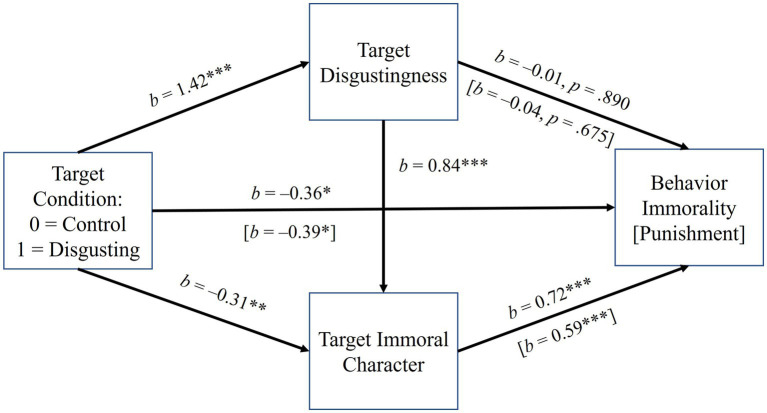
The effect of target condition on behavior immorality and punishment in Experiment 1, serially mediated by target disgustingness and immoral character. Coefficients are unstandardized. The serial indirect paths to behavior immorality and punishment through target disgustingness and immoral character were both positive and significant (*p*s < 0.05). As with the direct effect of condition, the indirect paths to behavior immorality and punishment through immoral character alone were significant (*p*s < 0.05) and negative, suggesting inconsistent mediation. Analyses controlled for violation type.

The last exploratory models [PROCESS (Hayes, 2017), Model 6, 10,000 bootstrap replications] are presented here. In two separate models, behavior immorality and punishment were, respectively, predicted by condition, with mediation running serially through target disgustingness and immoral character (see Figure 2). Condition (0 = control, 1 = disgusting) predicted target disgustingness (*p* < 0.001) and disgustingness predicted immoral character (*p* < 0.001). Target immorality predicted both behavior immorality and punishment, *p*s < 0.001. However, in both models, the direct effects of condition *negatively* predicted target immorality (*p*s = 0.004) and *negatively* predicted behavior immorality and punishment, respectively, *p*s = 0.029 and 0.013. For behavior immorality [punishment], indirect effects were significant and *negative* through target immorality alone (condition → target immorality → behavior immorality: *b* = −0.22, 95% CI = −0.39, −0.07) (condition → target immorality → punishment: *b* = −0.18, 95% CI = −0.34, −0.05) but significant and positive serially through target disgustingness and immorality (condition → target disgustingness → target immorality → behavior immorality: *b* = 0.86, 95% CI = 0.53, 1.26) (condition → target disgustingness → target immorality → punishment: *b* = 0.71, 95% CI = 0.39, 1.08).

### 2.3 Discussion

Overall, we found support for our primary hypothesis that the target we expected to be seen as disgusting (and which was rated as more disgusting) would be viewed as more immoral. However, there was no total effect of the target type of ratings of the immorality of the behavior. Similarly, there was no total effect of target type on the desire to punish the target. This was puzzling because of the high correlations of ratings of target immorality (i.e., character) with both behavior immorality and punishment. Further analyses revealed indirect effects suggesting that the behavior of the more (vs. less) disgusting target *was* rated as more immoral and that the more (vs. less) disgusting target was more deserving of punishment, statistically mediated by perceived disgustingness, which drove higher ratings of target immorality (i.e., serial mediation) before impacting behavior immorality and punishment. Yet, once controlling for these relationships, indirect (through character alone) and direct effects of condition on behavior immorality and punishment were significant and reversed in sign, suggesting suppression. Experiment 4 probes and discusses possible reasons for this, and further discussion is included in the General Discussion section.

## 3 Experiment 2

### 3.1 Methods

#### 3.1.1 Participants

Participants (*n* = 238; *M*_age_ 19.51, *SD* = 1.37; 164 females, 73 males, one “other” or preferred to not report) were a convenience sample of students from a large Midwestern university who received partial course credit for participation. Our goal was to collect at least 200 participants but to collect as much data as possible over the course of 1.5 semesters. Self-identified racial/ethnic categories were White/European American (*n* = 88), Black/African American (*n* = 28), Hispanic/Latino(a) (*n* = 35), Asian/Asian American (*n* = 72), and mixed/other (*n* = 15). On the same measure of ideology used in Experiment 1, the mean was 3.22, *SD* = 1.16.

#### 3.1.2 Procedure and measures

Participants read two separate vignettes that described “Andrew” and “Nathan,” who worked (counterbalanced method factor) as customer service representatives or quality control supervisors (counterbalanced order). After a job-related comprehension check, participants read (in counterbalanced order) about the targets’ habits, which we expected would be perceived as disgusting (e.g., enjoying the smell of feces) or probably unusual, but not disgusting (e.g., obsessively collecting and discarding mundane things). After completing a habit-related comprehension check and filler questions, participants learned (counterbalanced order) that one target behaved immorally (urinating on a fence outside an elementary school playground where children were present) and the other prosocially (buying dinner for and eating with a penniless stranger). The combination of target type (disgusting vs. odd) and behavior (moral violation vs. prosociality) is labeled “target/behavior pairing.”

Dependent measures differed as a function of behavior condition. Unless otherwise mentioned, items were measured on a scale from 1 = not at all to 7 = extremely. In the violation and prosocial conditions, respectively, character was measured with two and four items (immoral, evil, *r* = 0.58; moral, good, kind, caring, *α* = 0.93), behavior immorality and morality with four and five items (wrong, immoral, bad, forbidden, *α* = 0.88; correct, moral, kind, good, heartwarming, *α* = 0.87), and punishment/praise with a single item (1 = no punishment/praise at all, 7 = a lot of punishment/praise). Two items checking the manipulation (disgusting, sick, *r* = 0.76) were presented for the violating target only as a result of a programming error. Finally, participants rated, along with other emotions, how disgusted and grossed out they felt (*r* = 0.85) and provided demographic information.

### 3.2 Results

**Figure 3:**
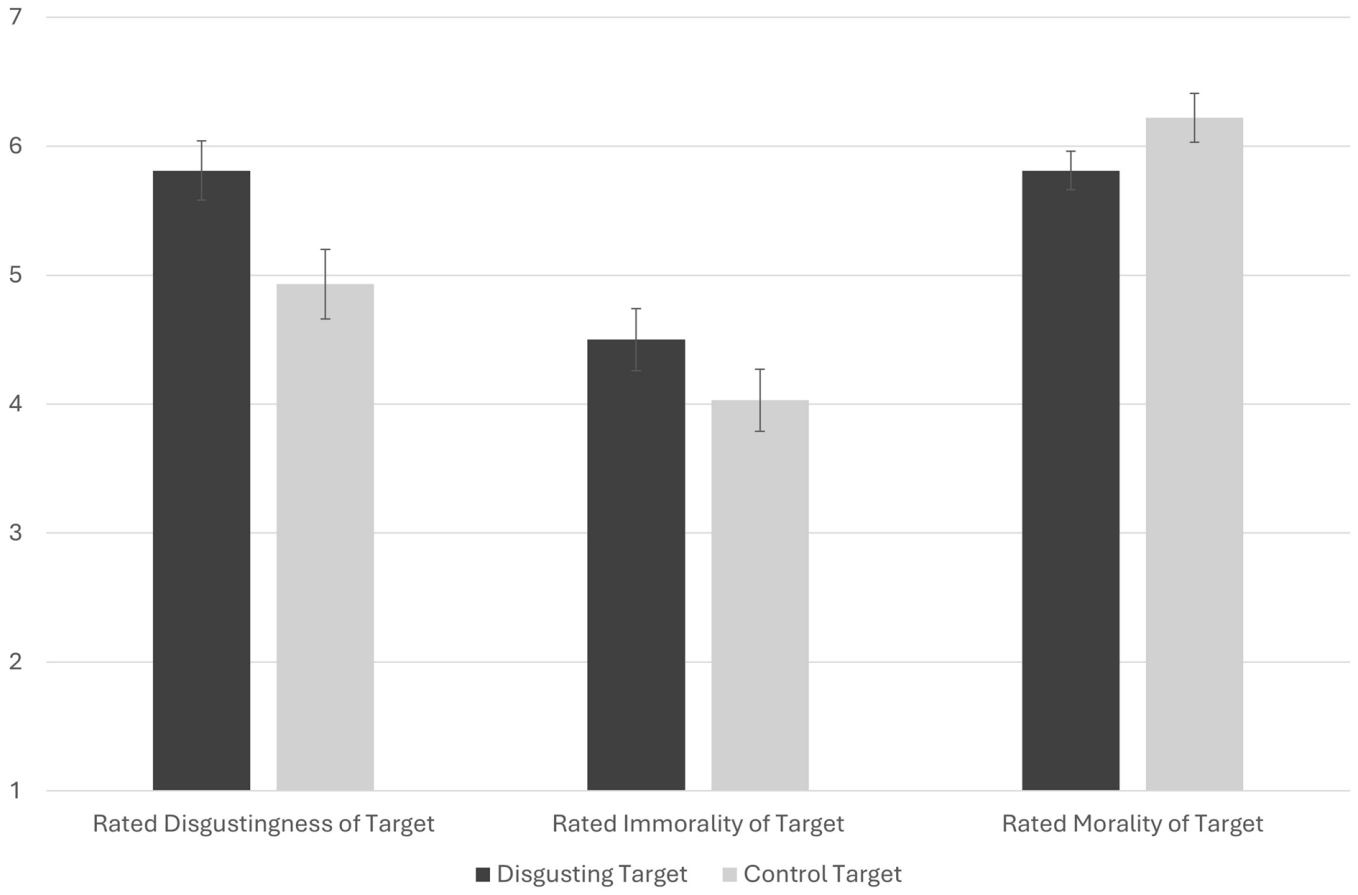
The effect of target condition on rated disgustingness (Violation Condition Only), immorality (Violation Condition Only), and morality (Prosocial Condition Only) in Experiment 2. Mean differences for all depicted variables were significant, *p*s < 0.012. Error bars are 95% confidence intervals. Variables are on seven-point scales.

The degrees of freedom for all between-participants ANOVA analyses was 1,234. Checking the manipulation, after committing a violation and regardless of whether the violation (vs. prosocial act) came first or second (*p* = 0.160; interaction *p* = 0.954), the disgusting target was seen as more disgusting (*M* = 5.81, 95% CI = 5.58, 6.03) than the odd target (*M* = 4.93, 95% CI = 4.66, 5.20), *F* = 23.08, *p* < 0.001, *d* = 0.64 (see Figure 3). Suggesting that participants’ reported feelings of disgust were not driving results; participants felt equally disgusted whether the disgusting or odd target committed a violation (*p* = 0.914; interaction *p* = 0.175). However, we note that participants’ reports of experiencing disgust were reported after reading and rating both vignettes, and people felt more disgusted overall when they read about the moral violation after rather than before the prosocial act, *p* < 0.001.

Replicating Experiment 1 and supporting our primary hypothesis, when committing a violation, people thought the disgusting target (*M* = 4.50, 95% CI = 4.26, 4.75) was more immoral than the odd target (*M* = 4.03, 95% CI = 3.79, 4.28), *F* = 6.64, *p* = 0.011, *d* = 0.35. People also thought the immoral target was more immoral when the violation (vs. prosocial behavior) was presented first, *p* = 0.047. However, the interaction of target/behavior pairing with order was not significant, *p* = 0.246. When behaving prosocially, people thought the disgusting target (*M* = 5.81, 95% CI = 5.66, 5.97) was less moral than the odd target (*M* = 6.22, 95% CI = 6.03, 6.41), *F* = 10.57, *p* = 0.001, *d* = 0.43. Neither order (*p* = 0.218) nor the interaction (*p =* 0.528) was significant.

As in Experiment 1, the *bad behavior* of the disgusting (vs. odd) target was not viewed as significantly more immoral (*p* = 0.389), other *p*s > 0.245. Similarly, although the disgusting (vs. odd) target was rated as descriptively more punishable, this effect was not significant, *p* = 0.088; other *p*s > 0.734. Yet, correlations of disgustingness and immoral character were both significantly positively correlated with behavior immorality and punishment, *r*s > 0.51, *p*s < 0.001. Similarly, the good behavior of the disgusting (vs. odd) target was not rated as less moral (all *p*s > 0.244) or praiseworthy (all *p*s > 0.139) even though moral character was positively and significantly correlated with behavior morality and praise (*rs* = 0.66 and 0.31, *p*s < 0.001).

**Figure 4:**
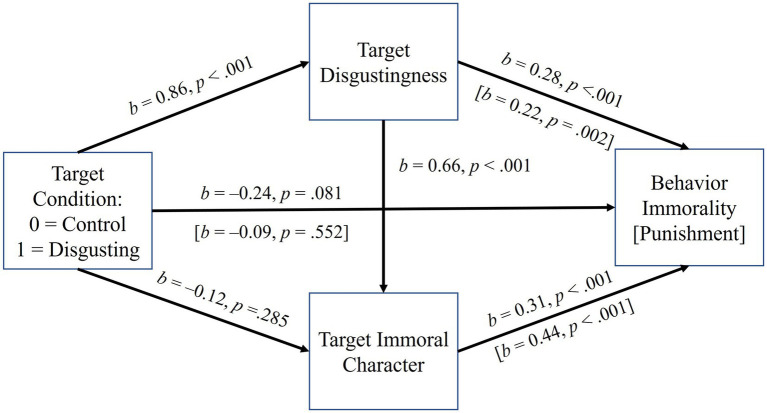
The effect of target condition on behavior immorality and punishment in Experiment 2, serially mediated by target disgustingness and immoral character. Coefficients are unstandardized. The indirect paths to behavior immorality and punishment through target disgustingness alone and serially through disgustingness and immoral character were both positive and significant (*p*s < 0.05). The indirect effects through immoral character alone, although negative in sign, were not significant, *p*s > 0.05. Analyses controlled for presentation order (i.e., whether transgressive or prosocial behavior was evaluated first).

Four mediation models were therefore examined. The first two duplicated the final exploratory analyses from Experiment 1, examining the effects of condition (0 = odd target immoral, 1 = disgusting target immoral) on behavior immorality (i.e., for the transgressive target’s behavior) and punishment serially through perceived disgustingness and immoral character, controlling for presentation order (see Figure 4). Positive indirect paths between condition and behavior immorality and punishment were found through disgustingness alone (condition → target disgustingness → behavior immorality: *b* = 0.23, 95% CI = 0.10, 0.42; condition → target disgustingness → punishment: *b* = 0.18, 95% CI = 0.02, 0.39), and serially through disgustingness and immoral character, condition → target disgustingness → target immorality → behavior immorality: *b* = 0.17, 95% CI = 0.08, 0.29; condition → target disgustingness → target immorality → punishment: *b* = 0.24, 95% CI = 0.0.12, 0.41. Thus, although no total effects emerged, indirect effects suggested that the disgusting (vs. odd) target’s *behavior* was also viewed as more immoral and punishable (i.e., because the target was viewed as more disgusting, which also made them seem more immoral). As in Experiment 1, direct effects (e.g., on immoral character, behavior, and punishment) and indirect effects through character only were reversed in sign, hinting at suppression (see the OSM for indirect effect coefficients). However, unlike Experiment 1, none of these effects were significant.

**Figure 5:**
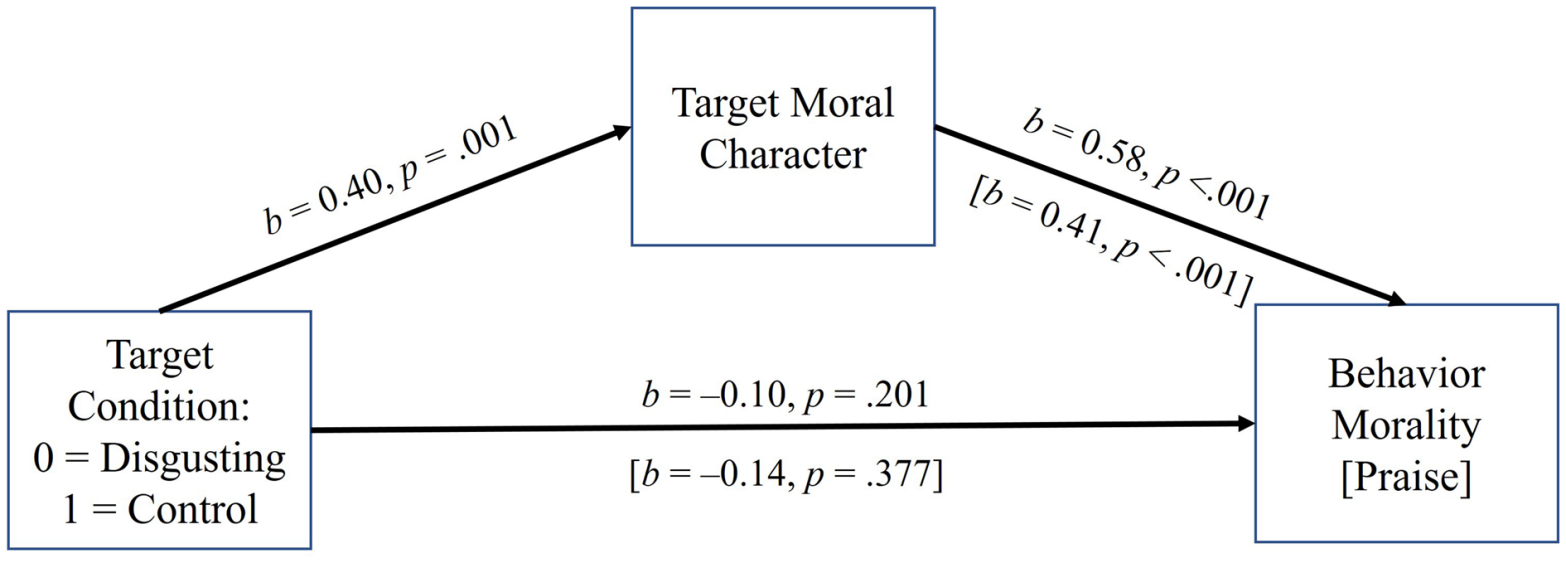
The effect of target condition on behavior morality and praise in Experiment 2, mediated by moral character. Coefficients are unstandardized. The indirect paths to behavior morality and praise through moral character were both positive and significant (*p*s < 0.05). Analyses controlled for presentation order (i.e., whether transgressive or prosocial behavior was evaluated first).

The third and fourth analyses examined the indirect effects of condition (0 = disgusting target prosocial, 1 = odd target prosocial), controlling presentation order, on behavior morality and praise (see Figure 5). Both indirect effects were significant, suggesting that because the disgusting target was less moral, their behavior was less moral and praiseworthy: behavior morality *b* = 0.23, 95% CI = 0.09, 0.37; praise *b* = 0.16, 95% CI = 0.05, 0.34. The direct effects of condition were reversed in sign, hinting at suppression, but were not significant, *p*s > 0.200.

### 3.3 Discussion

Experiment 2 again confirmed our hypotheses, this time in relation to the targets’ bad *and good* character. That is, targets who were described as having seemingly more (vs. less) disgusting habits were viewed as having more immoral character (when their behavior was counternormative) and as having *less moral* character (when their behavior was prosocial). In addition, as in Experiment 1, there were no total effects of target type on behavior immorality or desired punishment (for bad behavior). When evaluating good behavior, there were no total effects of target type on behavior *morality* or desired *praise.* Yet, in both cases, indirect effects—for bad behavior, serially through perceived disgustingness and immoral character for good behavior, through moral character alone—emerged, which showed that the manipulation of target type did impact perceptions of the badness/goodness of the behaviors and the desire to blame/praise the targets. This replicated findings from Experiment 1 and extended them further (i.e., replicating a similar pattern when targets behaved prosocially). We examine possible causes of these effects in Experiment 4.

## 4 Experiment 3

### 4.1 Methods

#### 4.1.1 Participants

Participants were 302 people (*M_age_* = 36.16, *SD* = 11.64; 133 females, 165 males, four reported other/prefer not to say) recruited from AMT and paid a small fee for their participation. Self-reported racial/ethnic identities were White/European American (*n* = 213), Black/African American (*n* = 17), Hispanic/Latino(a) (*n* = 20), and mixed/other (*n* = 22). Ideology was not assessed in Experiment 3 or Experiment 4.

#### 4.1.2 Procedure and measures

After providing consent, participants read about “Nathan,” an 8-year-old described as typical but who also had habits that some might consider disgusting (even if not completely atypical for a young child, such as eating boogers) or odd (e.g., loving vegetables and disliking sweets). Next, participants learned that Nathan had recently misbehaved at school (e.g., drawing on a desk; see SOM) before rating him on negatively (*α* = 0.74; naughty, a brat, and unfriendly) and positively valenced character traits (*α* = 0.83; kind, obedient, and intelligent) and behavior (*α* = 0.72; naughty, immoral, atypical, and bad) (1 = not at all, 9 = completely). Participants then estimated how frequently Nathan misbehaves in the classroom (1 = *almost never*, 7 = *very often*) and how much punishment he deserved for his behavior (0–8 min in “timeout”). Finally, participants rated their own emotions, including disgusted and grossed out (*r* = 0.79), before providing demographic information.

### 4.2 Results

**Figure 6:**
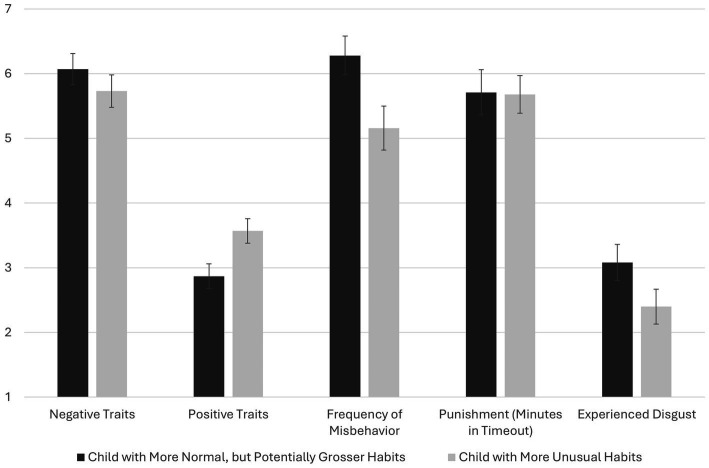
The effect of target condition on negative traits, positive traits, frequency of misbehavior, punishment, and participants’ self-reported disgust in Experiment 3. With the exception of participants’ self-reports of experienced disgust (*p* < 0.001), tests of mean differences control for experienced disgust. Mean differences were significant for positive traits and frequency of misbehavior, *p*s < 0.001. Controlling experienced disgust, mean differences were not significant for negative traits (*p* = 0.442) or punishment (*p* = 0.674). Presented means are not adjusted for the covariate. Error bars are 95% confidence intervals. Except for punishment, which was measured on a scale from 0 to 8 (minutes assigned to “time out”), variables were measured on a seven-point scale.

Although they did not feel very disgusted, participants in the disgusting child condition (*M* = 3.08, 95% CI = 2.80, 3.36) felt more disgusted than those in the unusual child condition (*M* = 2.40, 95% CI = 2.13, 2.67), *t*(300) = 3.46, *p* < 0.001, *d* = 0.40 (see Figure 6). Thus, subsequent analyses controlled for self-reported disgust. Reported means are unadjusted.

Although participants rated the disgusting child (*M* = 6.07, 95% CI = 5.83, 6.31) as slightly more “bad” (i.e., naughty, bratty, and unfriendly) than the unusual child (*M* = 5.73, 95% CI = 5.48, 5.99) when felt disgust was not controlled (*p* = 0.058, *d* = 0.22), this effect attenuated when controlling felt disgust, which was a significant covariate (*p* < 0.001), *F*(1, 299) = 0.59, *p* = 0.442. However, even controlling felt disgust (*p* < 0.001), the disgusting child (*M* = 2.87, 95% CI = 2.68, 3.06) was seen as significantly less “good” (i.e., kind, obedient, and intelligent) than the unusual child (*M* = 3.57, 95% CI = 3.38, 3.77), *F*(1, 299) = 34.49, *p* < 0.001, *d* = 0.58. Controlling for self-reported disgust (*p* < 0.001), participants also indicated that the disgusting child (*M* = 6.28, 95% CI = 5.98, 6.58) was more frequently disobedient than the unusual child (*M* = 5.16, 95% CI = 4.82, 5.49), *F*(1, 299) = 40.97, *p* < 0.001, *d* = 0.56 (Figure 6).

Controlling for self-reported disgust (*p* < 0.001), there was no effect of condition on behavior immorality (*p* = 0.409), although similar to negative character traits, the test that was not adjusted for felt disgust was significant, directionally suggesting that the disgusting (*M* = 5.83, 95% CI = 5.58, 6.07) (vs. unusual; *M* = 5.46, 95% CI = 5.22, 5.70) child’s behavior was viewed as somewhat more immoral, *p* = 0.037, *d* = 0.24. Finally, regardless of whether self-reported disgust was controlled (*p* = 0.004), the disgusting child (*M* = 5.71, 95% CI = 5.36, 6.06) was not rated as more punishable (assigned minutes in “time out”) than the odd child (*M* = 5.68, 95% CI = 5.39, 5.97), *p* = 0.674.

**Figure 7:**
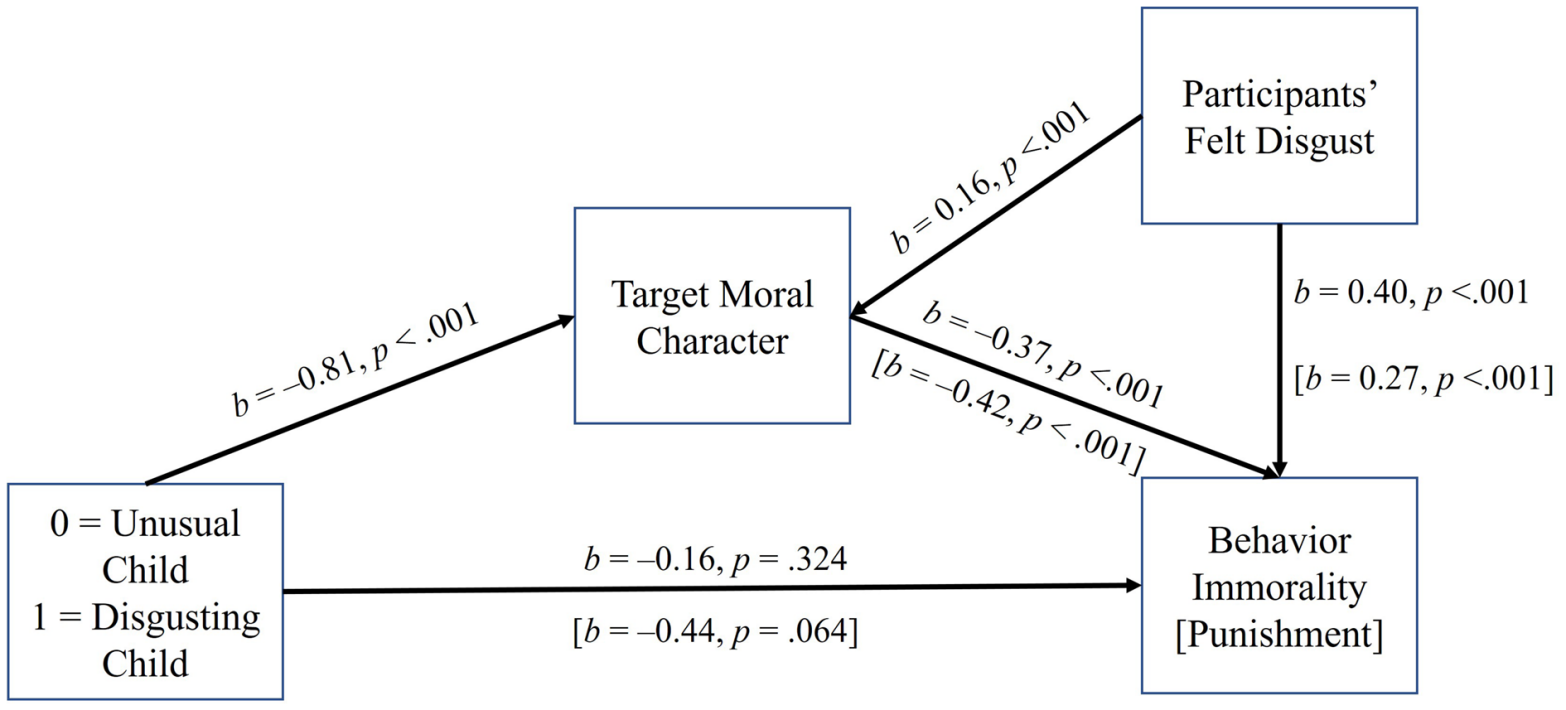
The effect of target condition on behavior immorality and punishment in Experiment 3, mediated by moral character. Coefficients are unstandardized. The indirect paths, controlling for participants’ self-reported disgust, to behavior immorality and punishment through moral character were both positive and significant (*p*s < 0.05).

Judgments of the child’s good character—which was responsive to condition when controlling felt disgusted—were subsequently explored as a mediator between condition (0 = odd, 1 = disgusting) and behavior immorality and punishment, controlling for self-reported disgust (see Figure 7). The indirect effects on behavior immorality (*b* = 0.30, 95% CI = 0.16, 0.46) and punishment (*b* = 0.34, 95% CI = 0.17, 0.56) were both significant, suggesting that because the disgusting (vs. unusual) child’s character was not as good, their behavior was more immoral and punishable. The direct effects of condition on behavior judgments (*p* = 0.324) and punishment (*p* = 0.064) were both negative, hinting once again at mixed effects, although neither path was significant. Thus, similar to Experiments 1 and 2, the belief that a disgusting (vs. unusual) person—in this case, a child—was less “good” (e.g., obedient) indirectly led to their behavior seeming more immoral and punishable even while direct effects of condition hinted at the idea that the disgusting (vs. unusual) child’s behavior was *less* immoral and punishable.

### 4.3 Discussion

Overall, the results of Experiment 3 were consistent with those of Experiments 1 and 2 and supportive of our primary hypothesis. That is, a child described in ways that some people would find disgusting (e.g., eating boogers and ear wax, digging through trash and eating recovered, half-eaten food) vs odd (e.g., preferring vegetables more than hot dogs, cheese, or sweets) was viewed less positively on traits such as kindness, obedience, and intelligence. The same plausibly more disgusting (vs. odd) child was also rated as one who misbehaved more frequently in the classroom. Although the more disgusting (vs. odd) child was rated as slightly higher in negative traits, and their behavior as more antisocial, these effects were not significant when controlling for participants’ reports of their own experienced disgust, which were also higher in the disgusting (vs. odd) child condition. Similarly, the time participants believed that the child should be assigned to a “time out” (i.e., punishment) for their behavior was not significantly impacted by the condition (i.e., no total effect of condition).

Similar to Experiments 1 and 2, we then explored whether positive moral character—which was significantly impacted by target condition when controlling felt disgusted—statistically mediated beliefs about behavioral immorality and punishment. Indirect effects were significant in both cases, suggesting the behavior of the more disgusting child *was* viewed as worse and more deserving of punishment, to the extent that this child’s character was seen less positively. This finding is quite similar to the effects found in Experiments 1 and 2. Experiment 4 was therefore conducted to explore one possibility for why these effects may have emerged.

## 5 Experiment 4

### 5.1 Methods

Participants were 290 people from AMT (*M*_age_ = 36.89, *SD* = 12.14; 133 females, 155 males, two reported other or preferred not to disclose). Beyond age and gender, no other demographic information was collected. Target descriptions were identical to Experiment 1. After reading about “John” and his plausibly more disgusting (vs. more typical) habits, participants rated his disgustingness before rating how *likely* it would be that John would engage in eight different immoral behaviors (*α* = 0.92; see SOM for a description) and how surprised they would be if he did behave in these ways (*α* = 0.89) on the same scale. Participants then rated John’s immoral character using a single item, rated how immoral each behavior would be if John performed it (*α* = 0.89), and rated how disgusted and grossed out they felt (*r* = 0.90). The same response scale (1 = not at all, 7 = extremely) was used for all measures.

### 5.2 Results

Controlling for the greater disgust participants felt when evaluating the disgusting (vs. average) target, participants rated the disgusting target as more disgusting and immoral, thought it was more likely that he would commit immoral acts, responded that they would be less surprised if he did, and indicated that the behaviors would be more immoral. Thus, unlike Experiments 1–3, each of the variables, including behavior immorality, was consistent with expectations. See Table 1 for means, 95% CIs, *t-*test values, significance levels, effect sizes, and correlations of each variable with self-reported disgust (see the SOM for the full correlation matrix).

**Table 1:** Descriptive and inferential statistics for Experiment 4.

	Disgusting target	Average target					
	*M* [95% *CI*]	*M* [95% *CI*]	*t*(288)	*p*_1_	*p*_2_	*D*	*r*
Self-reported disgust	4.06 [3.74, 4.38]	2.78 [2.49, 3.08]	5.74	<0.001	*—*	0.68	*—*
Disgustingness	5.66 [5.46, 5.86]	2.15 [1.87, 2.44]	20.11	<0.001	<0.001	2.36	0.51
Immoral character	3.66 [3.37, 3.95]	2.74 [2.44, 3.04]	4.40	<0.001	0.023	0.52	0.43
Behavior likelihood	3.54 [3.32, 3.76]	2.77 [2.52, 3.01]	4.68	<0.001	0.025	0.55	0.49
Surprise	4.42 [4.21, 4.63]	5.39 [5.20, 5.59]	6.60	<0.001	<0.001	0.78	−0.18
Behavior immorality	6.08 [5.96, 6.19]	5.80 [5.64, 5.96]	2.79	0.006	0.005	0.32	0.02

*p*_1_ and *p*_2_ are *p* levels without controlling for and after controlling for felt disgust; *r* is the correlation between each variable and self-reported disgust.

Next, we focused our attention on exploring two initially puzzling questions that helped motivate this final study. The first was why, since target disgustingness was positively associated with greater immorality, using it as a mediator between condition and immoral character would reveal that disgusting (vs. average/odd) targets seem both more immoral (indirect effect) and less immoral (direct effect). Second, we wanted to probe why, if being viewed as more disgusting and immoral (or less moral) are both indirectly associated with behavior being viewed as more immoral and punishable (accompanied by a variety of mixed and opposing direct and indirect effects), no total effects of condition on behavior and punishment would be found.

Correlations among measures in this study provide some clues to each of these questions. Specifically, perceived disgustingness and immorality were positively correlated with the perceived likelihood of bad behavior (respectively, *r*s = 0.55 and 0.64, *p*s < 0.001) and negatively correlated with expected surprise if the targets behaved badly (respectively, *r*s = −0.35 and − 0.24, *p*s < 0.001). Yet, likelihood was *negatively* correlated (*r* = −0.17, *p* = 0.003), and surprise was *positively* correlated (*r* = 0.12, *p* = 0.038) with the badness of behavior.

**Figure 8:**
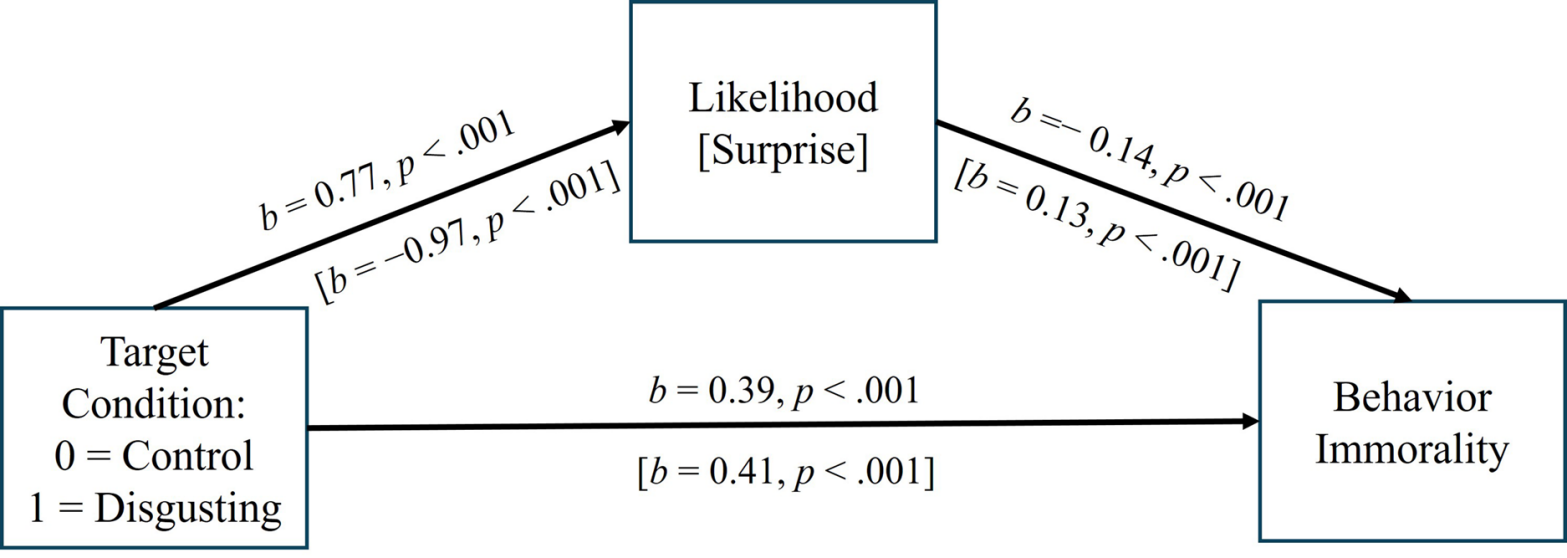
The effect of target condition on behavior immorality in Experiment 4, mediated by likelihood and surprise. Coefficients are unstandardized. The indirect paths were both negative and significant (*p*s < 0.05), opposite in sign from the total (and direct) effects.

These correlations suggest that when it seems less likely and more surprising that a person would do something bad, the bad behavior seems more immoral. Reasoning in reverse, it seems possible that when a person behaves badly, if they are perceived as disgusting (vs. average/odd), their bad behavior will seem less surprising and more expected, each of which suggests their behaviors are *less* immoral even though the people themselves seem *more* immoral. Yet, paradoxically, if the behaviors are less immoral, so should the person be. Two separate mediation models were examined to test these ideas. In each, we used condition (0 = average target, 1 = disgusting target) to separately predict behavior likelihood and surprise (mediators) and behavior immorality, with the mediators also predicting behavior immorality (see Figure 8). In the first model using the likelihood that the target would behave in various immoral ways, the indirect effect of condition was significant and negative in sign (*b* = −0.11, 95% CI = −0.19, −0.04), suggesting the behaviors would be *less* immoral if performed by the more disgusting target (i.e., an effect opposite from the total effect). The direct effect of condition on behavior immorality was also significant, but in the opposite direction (i.e., consistent with the total effect). Similarly, the indirect effect through surprise was significant and negative in sign (*b* = −0.13, 95% CI = −0.23, −0.04), again suggesting the behaviors would be *less* immoral if performed by the more disgusting target. And again, the direct effect of condition was significant, but in the opposite direction. Conducting the same analyses, but controlling for how disgusted/grossed out participants felt, revealed the same effects.

### 5.3 Discussion

Overall, the results of Study 4 were consistent with those of earlier studies. That is, participants rated a target we expected to be seen as more disgusting (and who was rated as more disgusting than a control target) as more immoral and likely to behave in a variety of immoral ways. Participants also indicated that they would be less surprised if this same target (vs. control) behaved in these ways. In a departure from earlier studies, however, they also rated the behaviors themselves as more immoral when they imagined the more disgusting performing them. Each of these analyses controlled for the higher disgust participants reported feeling when exposed to the more disgusting (vs. average) target.

One goal of this experiment was to replicate Experiments 1–3 further, using a situation where the target had not actually done anything wrong, to see if people would still view the target as more immoral, absent any behavioral evidence of their immorality. We confirmed this hypothesis. However, a second goal was to examine how *surprising* it would be for the target to behave in immoral ways, as we suspected that to the extent that participants viewed behavior as more likely and less surprising if performed by the target might tend to make the behaviors *indirectly* seem less immoral. By way of analogy, first imagine that a person, who has been convicted more than once of felony assault, kicks a friendly stray dog. Now, imagine a person one would expect to be a paragon of virtue, such as a religious leader, behaving in the same way. Either way, the behavior seems reprehensible. Yet, in our view, the behavior somehow seems worse when enacted by someone we would not expect to behave this way.

Carrying this reasoning forward, the felon (vs. religious leader) might be viewed as possessing more immoral character when they have behaved badly, in part because this behavior is more expected from them and may be a better indicator of their core character. Because of this, they might also be seen as more deserving of punishment for what they have done. Yet, at the same time, the behavior itself somehow seems worse when the religious leader does it, which should also translate into a desire to apply relatively greater punishment, at least to the extent that worse behavior deserves greater punishment in the minds of the evaluator. Correlations between how likely it would be for the targets to behave in these ways and how surprised participants would be if the targets behaved in these ways with how immoral the behaviors would be if performed by the targets seemed to confirm these ideas, as did additional tests of mediation. That is, each of these latter analyses seemed to suggest, statistically at least, that the behaviors would simultaneously be more *and* less immoral if performed by a more (vs. less) disgusting target.

## 6 General discussion

Prior research has suggested that disgust not only emerges in response to some moral violations (Rozin et al., 1999; Horberg et al., 2009; Giner-Sorolla et al., 2012) and character judgments (Giner-Sorolla and Chapman, 2017) but can also *influence* responses to moral violations (e.g., Schnall et al., 2008; Eskine et al., 2011), perhaps even making conventional (i.e., non-moral) violations appear to violate moral principles (e.g., Chapman and Anderson, 2014). However, the evidence for these latter propositions has been mixed, with recent articles suggesting that incidental disgust is not systematically associated with moral judgments (Landy and Goodwin, 2015; but see Schnall et al., 2015).

In this study, we set out to examine what we believe is a novel question related to disgust and moral judgment: Similar to the idea that dirty people seem less trustworthy (Rottman et al., 2020), we wanted to examine whether people with *habits* that many people might find disgusting (i.e., people who *seem* disgusting) are viewed as having more immoral character, as compared with more “ordinary” targets. Support was found for this primary hypothesis in four experiments.

In our view, this is an interesting question because when considering the range of human behaviors and preferences, “regular” people are likely to sometimes behave in ways that others would consider disgusting. This is certainly true for people’s private, unobserved behaviors. Still, some people do not care whether their actions seem disgusting, and others may be “caught” doing something others view as disgusting, even when trying to hide it. Similarly, even if a social target is not “doing” anything observers would label as disgusting, there may be something about them that nevertheless evokes disgust (e.g., Giner-Sorolla et al., 2012; Watanabe and Laurent, 2020), which could prompt moral character evaluations if that dimension of judgment is relevant.

However, in addition to this primary question regarding the perception of moral character based only on a target’s habits, we were also interested in related questions such as whether disgust would influence evaluations of behavior morality and beliefs that people deserve punishment (or praise). Although we had expected similar results for ratings of behavior and the application of punishment, we found a more complicated set of results. That is, the pattern we found suggests that the behavior of disgusting (vs. average or odd) people might be viewed as both more and *less* immoral and punishable.

Experiment 1 established that a person with disgusting habits was viewed as more immoral than a person with average habits. In an exploratory statistical model, we also found that because one target was viewed as more disgusting than the other, which predicted them being rated as more immoral, their behavior was viewed as more immoral and punishable. However, after controlling for the effects of the targets’ disgustingness, direct effects suggested that the disgusting target was somewhat *less* immoral. Similarly, after controlling for target disgustingness, immoral character, or both, direct effects of condition suggested that their behavior was somewhat *less* immoral and punishable. Experiment 2 replicated and extended these effects, showing that even when the comparison target had *odd* (i.e., rather than average) habits, similar effects emerged on judgments of both immorality and morality. Similarly, the interesting but unpredicted and exploratory mediation effects found in Experiment 1 were essentially reproduced.

Experiment 3 further extended these effects, pitting a child with somewhat typical (for a child) but potentially disgusting habits against a child with habits that were certainly unusual but not likely to be perceived as disgusting. Even though the targets were children, and their potentially disgusting (to adults) behavior might be considered somewhat normative, college students believed that the disgusting (vs. odd) child was less “good” (e.g., obedient and intelligent) and probably behaved badly more frequently, even though effects on the child’s “bad” character were reduced after controlling for felt disgust. Once again, effects on behavior immorality and punishment only emerged indirectly through character evaluations.

Notably, in each of these studies, the targets being evaluated had also done something wrong (or prosocial in Experiment 2), with evaluations only following information about their bad behavior. Experiment 4, therefore, took a different approach, by describing targets and their habits, then asking participants how *likely* these targets would be to perform different immoral behaviors and how *surprising* it would be for them to behave in these ways. Following this, participants were asked a single question about how immoral the targets were and how immoral the behaviors would be if the targets performed them. Thus, although we referenced several types of bad behaviors and asked participants to consider the likelihood of participants behaving in these ways, the targets were never actually described as having done anything wrong. Despite this, based on the only information that was varied (i.e., the targets’ habits), the target that people rated as more disgusting was seen as more immoral.

Potentially interesting as a way of explaining the unpredicted indirect effects in Experiments 1–3 (and the lack of total effects of condition on behavioral immorality and punishment in these studies), the target rated as more disgusting was also rated as more likely to behave immorally, with participants also indicating that they would be less surprised if the more disgusting target behaved badly. These effects make sense, as one might think it is likelier and less surprising for a more (vs. less) immoral person to behave immorally. Yet, despite people rating the behaviors as more immoral when they imagined the more (vs. less) disgusting target performing them—which was not the case for ratings of the behaviors that targets were described as performing in Experiments 1–3—behavior likelihood was *negatively* correlated with behavioral immorality ratings, and surprise was *positively* correlated with behavioral immorality ratings. Mediation tests confirmed our speculative expectations given the results from Experiments 1–3: that people would view behaviors as both more *and* less immoral if performed by a more (vs. less) disgusting target, as a function of differences in how likely and surprising those behaviors would be.

More broadly, if a person’s behavior seems comparatively less surprising and more predictable because of who they are, this might decrease the extent to which those behaviors are seen as bad. That is, evaluations of behavior are not context-free (see Guan and Heine, 2022), and this may influence how behavior is evaluated. For example, when evaluating a person’s moral character and the only available information is their behavior (e.g., no information about past behaviors is available), judgments should rely almost exclusively on that behavior. However, when perceivers’ preexisting ideas about a target category influence beliefs about the morality of a target from that category (even when no previous and relevant behavioral evidence from that target is available), judgments of the badness of a behavior will likely rely not only on the behavior itself but on unfounded *beliefs about the person* who enacted it. Thus, when being compared to a target where no prior expectations are in place, overattributing immoral character because of some feature of a target might lead to a mixed set of evaluations when it comes to the desire to punish that target for what they have done.

We acknowledge that this idea is speculative, but the pattern that emerged in our data suggests it is a possibility worth considering, even if it is difficult to conclude anything about causality given the reliance of the present studies on statistical mediation methods. That is, experimental tests of this effect would be needed to draw firmer conclusions—not only about the present set of results, but about this effect more broadly. Yet, testing these ideas experimentally in the same design might be somewhat difficult to do convincingly because one way of conceptualizing the immorality of a target’s character essentially resides in what one might predict about that target’s future behavior. Nevertheless, we think the idea we are advancing here is interesting, and we hope that future research will address it further.

Some further limitations of this work should be considered. First, it is important to recognize that this research was conducted solely in the United States, using convenience samples consisting of mostly White/European Americans drawn from an online labor market and a midwestern university. Drawing more general conclusions about the relation of target disgust to moral judgment would require more diverse samples, both in racial/ethnic categories and in cultures/geographic locations. Second, and relatedly, although evolved disgust is probably a culture-free emotion (Curtis et al., 2011; Tybur et al., 2013) in that it may have similar effects on evaluation across cultures (Andersson et al., 2024) and people everywhere experience it in response to particular pathogens or stimuli, the extent to which particular behaviors or social categories *cause* a person to seem more disgusting is likely to vary across people, cultures, and contexts. Thus, even if the extent to which people see others as disgusting based on their behaviors or something else (Skinner and Hudac, 2017) has similar impacts on moral evaluations of those others, more research would be needed to firmly draw that conclusion. For example, although in some contexts, people might find it disgusting to consider a person eating food they recovered from a dumpster, the same behavior might be seen as not only understandable, but as necessary if the person was starving. Finally, the current research relied exclusively on vignettes, so further work must be done using other types of stimuli (e.g., photos, videos, and live interactions) and dependent measures (e.g., punishment paradigms, behavioral measures of approach/avoidance) to understand the real-world consequences of this effect, if they exist.

Still, beyond these caveats, the present research seems to suggest that people others believe are relatively disgusting will be perceived as relatively more immoral and more likely to behave immorally than people who are viewed as less disgusting. If confirmed using additional methods, this is likely to have important real-world consequences, particularly considering the frequency with which people do things that others might consider disgusting (even if the people evaluating others do similar disgusting things themselves). Although this work is only a start, we think that it can be built on easily and that this question represents an interesting direction for future research.

## Data availability statement

The datasets presented in this study can be found in online repositories. The names of the repository/repositories and accession number(s) can be found below: https://tinyurl.com/22juru46.

## Ethics statement

The studies involving humans were approved by the Office for the Protection of Research Subjects, University of Illinois at Urbana-Champaign. The studies were conducted in accordance with the local legislation and institutional requirements. The participants provided their online informed consent to participate in this study.

## Author contributions

SL: Conceptualization, Data curation, Formal analysis, Investigation, Methodology, Project administration, Resources, Supervision, Writing – original draft, Writing – review & editing. JL: Conceptualization, Investigation, Methodology, Writing – original draft.

## Footnotes

^1^ https://tinyurl.com/22juru46
